# Band Gap Engineering of Newly Discovered ZnO/ZnS Polytypic Nanomaterials

**DOI:** 10.3390/nano12091595

**Published:** 2022-05-08

**Authors:** Dejan Zagorac, Jelena Zagorac, Milan Pejić, Branko Matović, Johann Christian Schön

**Affiliations:** 1 Materials Science Laboratory, Institute of Nuclear Sciences “Vinča”, University of Belgrade, 11000 Belgrade, Serbia; jelena@vinca.rs (J.Z.); milan.pejic@yahoo.com (M.P.); mato@vinca.rs (B.M.); 2 Center for Synthesis, Processing and Characterization of Materials for Application in the Extreme Conditions “Cextreme Lab”, Institute of Nuclear Sciences, University of Belgrade, 11001 Belgrade, Serbia; 3 Nanoscale Science Department, Max Planck Institute for Solid State Research, 70569 Stuttgart, Germany

**Keywords:** ZnO/ZnS, polytypes, band gap, ab initio, semiconductors, nanostructured ceramics

## Abstract

We report on a new class of ZnO/ZnS nanomaterials based on the wurtzite/sphalerite architecture with improved electronic properties. Semiconducting properties of pristine ZnO and ZnS compounds and mixed ZnO_1−x_S_x_ nanomaterials have been investigated using ab initio methods. In particular, we present the results of our theoretical investigation on the electronic structure of the ZnO_1−x_S_x_ (x = 0.20, 0.25, 0.33, 0.50, 0.60, 0.66, and 0.75) nanocrystalline polytypes (2H, 3C, 4H, 5H, 6H, 8H, 9R, 12R, and 15R) calculated using hybrid PBE0 and HSE06 functionals. The main observations are the possibility of alternative polytypic nanomaterials, the effects of structural features of such polytypic nanostructures on semiconducting properties of ZnO/ZnS nanomaterials, the ability to tune the band gap as a function of sulfur content, as well as the influence of the location of sulfur layers in the structure that can dramatically affect electronic properties. Our study opens new fields of ZnO/ZnS band gap engineering on a multi-scale level with possible applications in photovoltaics, light-emitting diodes, laser diodes, heterojunction solar cells, infrared detectors, thermoelectrics, or/and nanostructured ceramics.

## 1. Introduction

Pristine zinc oxide (ZnO) and zinc sulfide (ZnS) are tetrahedrally coordinated binary A^II^B^VI^ compounds with many desirable properties for industrial applications. They both can crystallize in two main crystalline forms: the cubic sphalerite (or zincblende, 3C) and the hexagonal wurtzite (2H) polymorph, depending on the synthesis conditions. While ZnS is relatively abundant in nature, mainly as the mineral sphalerite [1,2,3], ZnO, although one of the most investigated compounds in materials science, is rarely found in its pure form, as the mineral zincite [1,2,4]. In the laboratory, both ZnS and ZnO can be synthesized in a variety of modifications, colors, and shapes (bulk, thin-film, nanowire, etc.), etc., depending on the synthesis conditions [5,6,7,8,9,10,11]; in addition, the effects of defects and dopants have been extensively investigated [12,13,14,15,16,17].

ZnO has been subject to extensive research activities due to its direct and wide band gap (3.44 eV at low temperatures and 3.37 eV at room temperature), large exciton binding energy, piezoelectricity, strong luminescence, strong nonlinear resistance, high thermal conductivity, availability of large single crystals, large nonlinear optical coefficients, small effective electron mass, the sensitivity of surface conductivity to the presence of adsorbed species, and other properties desirable for device applications [8,18]. ZnO has some advantages over GaN, another wide-band-gap semiconductor, which is widely used in optoelectronic applications: compared to GaN, ZnO exhibits high radiation hardness, which is important for applications in space or at high altitudes, and, furthermore, high-quality ZnO bulk single crystals can be produced. ZnO has a larger exciton binding energy (~60 meV vs. 25 meV in GaN), and the crystal-growth technology of ZnO is also much simpler, which has the potential to greatly lower the production costs of ZnO-based devices [8,19].

ZnS, as with ZnO, is a well-known wide-band-gap semiconductor from the II-VI family (3.6 eV at room temperature) and is a promising candidate for many technological applications as a versatile material with excellent optical and electrical properties, and a wide range of possible structures and morphologies, possessing chemical and thermal stability. Its applications include transparent conductors, visual displays, high-density optical memories, light-emitting diodes (LEDs), infrared windows, sensors, lasers, and use in electroluminescence, photoluminescence, and photocatalysis (when it is combined with other semiconductors or doped with transition metal ions). Applications of ZnS nanostructures include LEDs, dye-sensitized solar cells, with novel research of one-dimensional nanostructures focused on field-effect transistors, field emission, photodetectors, and sensors (gas, thermal, humidity, ion-sensors, and biosensors) [20,21,22].

The electronic structure of ZnO and ZnS can be modified via the replacement of oxygen by sulfur in ZnO or sulfur by oxygen in ZnS [23]. Recent studies of ZnO/ZnS heterostructures and heterojunctions with various morphologies have been reported [24,25,26,27,28,29,30]. These materials mostly exhibit core/shell structures and have found very wide technological applications such as solar cells, nonlinear optical devices, displays, infrared windows, optoelectronic devices, catalysis, biosensors, electronics, magnetism, mechanics, electrochemistry, and ceramics [31,32,33,34,35,36,37,38,39]. In this paper, we perform an in-depth ab initio-level theoretical study of band-gap engineering of ZnO/ZnS nanosized semiconducting phases.

## 2. Materials and Methods

The ab initio calculations were performed using the CRYSTAL17 package (University of Torino, Torino, Italy) [40,41], based on linear combinations of atomic orbitals (LCAO), while for the local optimizations, analytical gradients were employed [42]. The polytype structure candidates studied here had been generated in earlier work in the mixed zinc oxide-sulfide system ZnO_1−x_S_x_, (for x = 0, 0.2, 0.25, 1/3, 0.5, 0.6, 2/3, 0.75, 1), using the so-called Primitive Cell approach for Atom Exchange (PCAE) method [43]. In the PCAE method, the mixed polytypes for each composition had been generated by replacing the appropriate number of layers of oxygen atoms with sulfur atoms, keeping the reduction in crystal symmetry to a minimum in this process. This had been followed by a local minimization on the ab initio level using two different functionals (HSE (Heyd–Scuseria–Ernzerhof) and LDA (Local-density approximation)). In our previous studies on ZnO and ZnO/ZnS compounds, hybrid functionals had shown the best accuracy when computing the structural properties [11,43,44,45].

Nevertheless, for the band-structure calculations presented in this work, the use of additional hybrid functionals for comparison is of importance, and thus we re-optimized all structures using the PBE0 and the HSE06 hybrid functionals, before performing the band-structure calculations. Generally, using several different ab initio methods is highly useful to get some feeling for the quantitative validity of the results (especially in cases where no experimental data are available for comparison) [46,47,48]. The hybrid HSE06 (Heyd–Scuseria–Ernzerhof) exchange-correlation functional uses an error-function-screened Coulomb potential to calculate the exchange portion of the energy to improve computational efficiency [49], while the PBE0 functional mixes the Perdew–Burke–Ernzerhof (PBE) exchange energy and Hartree–Fock exchange energy in a 3:1 ratio, along with the full PBE correlation energy [50,51].

All-electron basis sets (AEBS) based on Gaussian-type orbitals (GTO) were used. In the case of Zn^2+^, a [*6s5p2d*] basis set was used as in refs. [44,52,53]. For O^2^^−^, a [*4s3p*] basis set was used as in refs [44,54,55]. For S^2^^−^, a [*5s4p1d*] all-electron basis set was used as in refs. [56,57], while their combination was used as in ref. [43]. A *k*-point sampling net of size 8 × 8 × 8 was used. For the analysis of the structures and their visualization, we used KPLOT [58] and the VESTA [59] program, and for the visualization of the band structures, the Xmgrace progam [60] was employed.

## 3. Results

### 3.1. Structural Investigation of ZnO/ZnS

Regarding the ab initio modeling and structure analysis, we first present the results for the pure ZnO and ZnS compounds, followed by the results for predicted polytypic nanostructures as alternatives, which might be present in the interface region of synthetic and natural ZnO/ZnS crystals, and of core–shell particles, heterostructures, and thin films. If one compares theory and experiment for pristine ZnO and ZnS compounds, our hybrid PBE0 and HSE06 density functional calculations are in good agreement with previous experimental data for the ZnO and ZnS phases (see supporting information, Table S1). Additional calculated structural data of all investigated nanostructured polytypes for pure ZnO and ZnS are compared to experimental data in the supporting information (Table S1). Although there exist no experimental data on ZnO polytypes, our present calculations concur with previous observations in the ZnS compound.

Apart from pristine ZnO and ZnS compounds, a large number of mixed ZnO_1–x_S_x_ compounds have been investigated for various compositions (x = 0.20, 0.25, 0.33, 0.50, 0.60, 0.66, and 0.75), where we consider a multitude of possible stable nanocrystalline polytypes for ZnO/ZnS compounds (2H, 4H, 5H, 6H, 8H, 9R, 12R, 15R, and 3C). To compare various polytypes with known wurtzite (2H) and sphalerite (3C) polymorphs in numerous mixed ZnO_1–x_S_x_ systems, a common supercell approach has been applied. However, one notices a potentially high level of distortion beyond the one enforced by the presence of both sulfur and oxygen atoms in the structure when creating large supercells of wurtzite and sphalerite to compare to the predicted polytype structures created by the PCAE method. In particular, the wurtzite-type may distort to the trigonal *P*3*m*1 (no. 156) space group, and the sphalerite structure can easily distort to the rhombohedral *R*3*m* (no. 160) space group. Furthermore, with a substitution of 25% or 75% of the sulfur atoms in ZnO, the symmetry of wurtzite could be reduced to reach a monoclinic symmetry (space group *Pm*, no. 6) and that of sphalerite could be reduced to reach the orthorhombic *Amm*2 (no. 38) space group, respectively.

In practice, when analyzing the structural behavior of the predicted polytypic structures in various mixed ZnO_1–x_S_x_ compounds, we noticed a lower level of structure distortion. In fact, all polytypes remain in the same space group regardless of sulfur/oxygen content, except for the 4H and 15R polytypes. Structural data of the 4H polytype in various ZnO/ZnS compositions are shown in Table 1. The 4H polytype has been observed in pure ZnS in the hexagonal *P*6_3_*mc* (no. 186) space group, which was confirmed by both hybrid calculations. It is expected to be observed in the same symmetry in the pure ZnO compound, while in the mixed ZnO/ZnS chemical systems, the symmetry is reduced to the trigonal *P*3*m*1 (no. 156) space group (Table 1). In the case of the 15R polytype, the only experimental observation is in the *R*3*mH* (no. 160) space group (Table 2). This polytype will remain undistorted in pure ZnO and various mixed ZnO_1–x_S_x_ compounds, except in the case of a 1:1 mixture of sulfur and oxygen. In the case of the ZnO_0.5_S_0.5_ compound, the 15R polytype is the energetically most favorable structure candidate among the polytypes and the symmetry is reduced to the monoclinic *Cm* (no. 8) space group. This will all have a strong effect on the electronic properties, which are investigated in detail in Section 3.2 below.

Moreover, we have performed additional calculations in order to further analyze and compare our calculated polytypic nanostructures with the earlier experimental and theoretical data in pure and mixed ZnO/ZnS compounds. In contrast to those calculations, where both atom positions and cell parameters were optimized, we have also used the experimentally observed wurtzite (2H) and sphalerite (3C) phases, as well as predicted 4H, 8H, and 12R polytypes, as starting points for additional optimization in the pure and mixed ZnO/ZnS systems. These optimizations were performed by fixing atomic positions in the idealized (high-symmetry) positions in the perfect structure, and only the unit cell parameters were allowed to relax. We note that this limited optimization does not affect the structure itself—which is not surprising—but it has a dramatic effect on the electronic properties and size of the band gap. Interestingly, we obtain a good agreement with band-gap measurements of core–shell ZnO/ZnS structures (as well as with those previous calculations that employed the same restrictions). In addition, the above structures were investigated for a possible influence on the size of the supercell and the position of the layers of the sulfur atoms in the structure (four types of layer arrangements, see supporting information, Table S14). We note that the greatest influence of the sulfur layer position is found for the distorted wurtzite structure, while the predicted polytypic structures are barely affected. However, all of these structural changes affect the band gap, which is investigated in detail in the text below.

### 3.2. Electronic Band Structure Calculations

**Figure 1:**
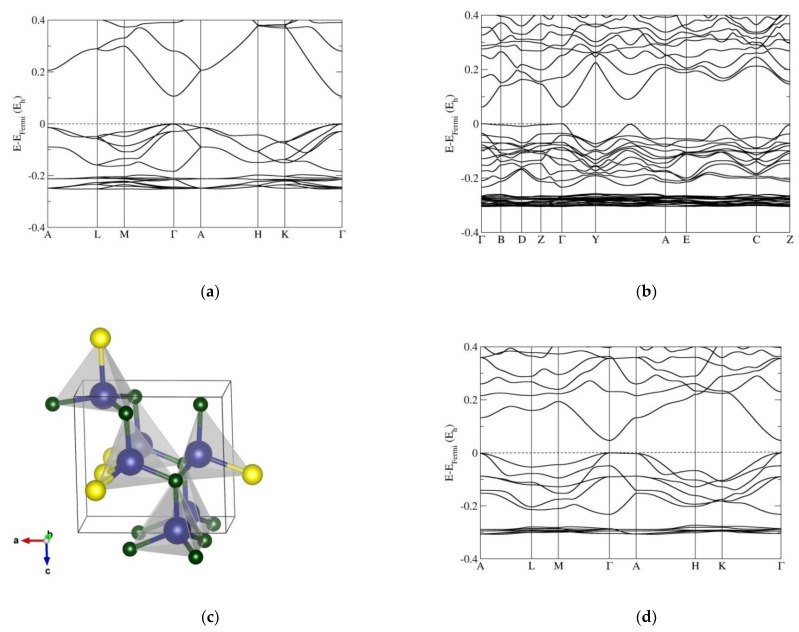

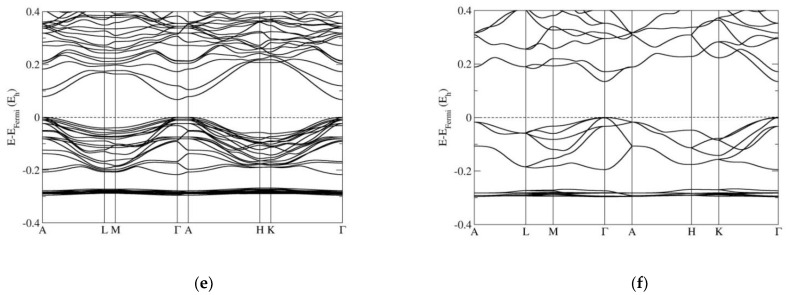
Band structures of the wurtzite (2H) phase computed using the HSE06 functional for different compositions: (**a**) pure ZnO with undistorted hexagonal (*P6_3_mc*) 2H phase; (**b**) ZnO_0.75_S_0.25_ compound with strongly distorted monoclinic (*Pm*) 2H phase; (**d**) ZnO_0.5_S_0.5_ compound with a slightly distorted 2H structure with trigonal (*P3m1*) symmetry; (**e**) ZnO_0.33_S_0.66_ compounds, again with a distorted 2H structure with space group *P3m1*; (**f**) pure ZnS compound with undistorted hexagonal (*P6_3_mc*) 2H phase. (**c**) shows the distorted crystal structure for x = 0.25. Blue, green, and yellow spheres correspond to Zn, O, and S atoms, respectively. Note that for ZnO_0.75_S_0.25_ and ZnO_0.25_S_0.75_ (in Figure S1 in the Supplementary Materials), the labels of the special points and paths of the Brillouin zones correspond to those of a monoclinic lattice, while in the other band structures, they correspond to those of a hexagonal lattice. Energies are given in Hartree (1 E_h_ = 27.211 eV).

The band structures and band gaps were computed for the nanostructured polytypes (2H, 4H, 5H, 6H, 9R, 12R, 15R) of the mixed compounds ZnO_1−x_S_x_ for those compositions x (0, 0.25, 0.33, 0.5, 0.67, 0.75, 1) that are compatible with the polytype, using both the PBE0 and the HSE06 functionals. For the experimentally observed wurtzite and sphalerite-type ZnO_1−x_S_x_ compounds with sulfur content (x), the band gaps are presented in Table S6 in the supporting information. Similarly, the calculated band gaps for the hexagonal 4H, 5H, 6H, and 8H, and rhombohedral 9R, 12R, and 15R predicted polytypes—for the different compositions—are presented in the supporting Tables S7 and S8, respectively. Figure 1 shows the band structures of the wurtzite (2H) phase under various ZnO/ZnS compositions; additional band structures for the 2H-type are provided in the supporting information, Figure S1.

The band structure calculations performed on the wurtzite (2H) phase ZnO_1−x_S_x_ (x = 0, 0.25, 0.33, 0.50, 0.66, 0.75, and 1) show a direct band gap at the Γ point of the Brillouin zone, especially noticeable for the pristine ZnO and ZnS compounds in agreement with band gap measurements (Figure 1). However, we note that with 25% sulfur substitution in ZnO (i.e., ZnO_0.75_S_0.25_), the 2H modification is strongly distorted to the monoclinic (*Pm*) symmetry, and the band gap is reduced (HSE06: E_g_ = 1.68 eV; PBE0: E_g_ = 2.30 eV). Furthermore, the separation between the top of the valence band (TVB) and the bottom of the conduction band (BCB) narrows along the Y-A direction, and the TVB is increased in other directions (EC, BDZ), too, thus creating the possibility that an indirect band gap might appear, (or so-called secondary band gap [45]).

A similar effect has been observed in the ZnO_0.25_S_0.75_ compound, but here, the band gap is increased (Figure S1 in the Supplementary Materials). In the case of the ZnO_0.66_S_0.33_ system, the band gap is further reduced (HSE06: E_g_ = 0.54 eV; PBE0: E_g_ = 1.16 eV), resulting in a narrow-gap semiconductor material (Figure S1). For the 1:1 sulfur/oxygen ratio (ZnO_0.5_S_0.5_), the band gap is larger than for x = 0.33, and continues to grow with sulfur content. The 2H structure is slightly distorted with trigonal (*P3m1*) symmetry, which has a hexagonal lattice (this also holds for the compositions x = 0.33 and 0.67), and the TVB is increased at the A point, or along the Γ-A direction, which makes it interesting from the point of future applications of 2H-ZnO_1−x_S_x_ as direct/indirect ZnO/ZnS semiconductors. A similar behavior is observed for the ZnO_0.66_S_0.33_, ZnO_0.5_S_0.5_, and ZnO_0.33_S_0.66_ 2H-compounds, and appears typical for the hexagonal polytypes.

**Figure 2:**
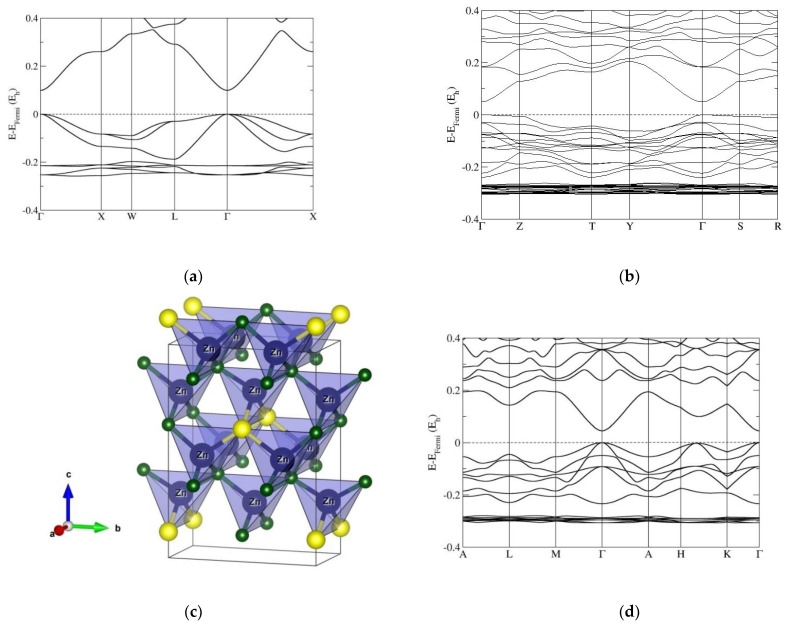

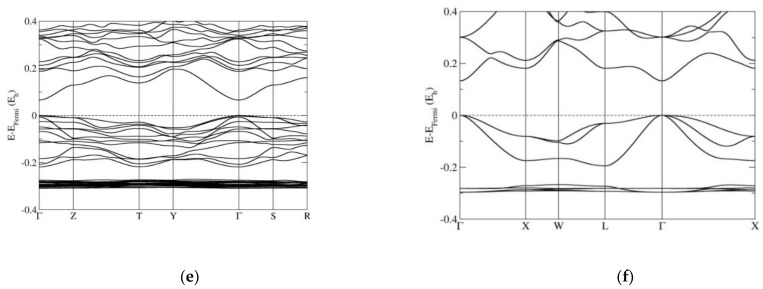
Band structures of the sphalerite (3C) phase computed using the HSE06 functional: (**a**) pure ZnO with undistorted cubic (*F*-43*m*) 3C phase; (**b**) ZnO_0.75_S_0.25_ composition with strongly distorted orthorhombic (*Amm*2) 3C phase; (**d**) ZnO_0.5_S_0.5_ with slightly distorted 3C structure with trigonal (*R3m*) symmetry; (**e**) ZnO_0.25_S_0.75_ compound, again with a strongly distorted orthorhombic 3C phase; (**f**) pure ZnS compound with undistorted cubic 3C phase. (**c**) depicts the distorted crystal structure for x = 0.25 sulfur substitution. Blue, green, and yellow spheres correspond to Zn, O, and S atoms, respectively. Note that the labels of the special points of the Brillouin zones correspond to those of an orthorhombic lattice (**b**,**e**), those in the case of trigonal 3C distortion correspond to those of a hexagonal lattice (**d**), while, in the other cases, the zones correspond to those of a cubic *fcc* lattice (**a**,**f**).

Band-structure calculations performed on the cubic sphalerite (3C) phase ZnO_1−x_S_x_ (x = 0, 0.25, 0.33, 0.50, 0.66, 0.75, and 1) also show a direct band gap at the Γ point of the Brillouin zone, most clearly seen in the pure ZnO and ZnS compounds (Figure 2). In the ZnO_0.75_S_0.25_ compound, the 3C structure is strongly distorted to the orthorhombic (*Amm2*) phase (Figure 2c); as a consequence, the band gap is reduced, and the highest energy state of the valence band at the Z and S point of the Brillouin zone is competing with TVB at the Γ point (Figure 2b). A similar effect has been observed in the ZnO_0.25_S_0.75_ compound; only the band gap is increased (Figure 2e). In the case of the ZnO_0.66_S_0.33_ 3C-modification, the band gap is further reduced, resulting in a narrow-gap semiconducting material (Figure S2), in a nonfavorable hexagonal symmetry, however. For the ZnO_0.5_S_0.5_ compound, the band gap is again larger than for the x = 0.33 case and continues to increase with further sulfur content x, as in the 2H polytype (Figure 2d). Here, the 3C structure is slightly distorted to a trigonal (*R3m*) symmetry, which exhibits a rhombohedral lattice. Moreover, the TVB and BCB along the H-K direction of the Brillouin zone are narrowed (i.e., indicating the possibility of the appearance of a secondary gap), observed also in other rhombohedral polytypes (such as 9R, 12R, and 15R).

**Figure 3:**
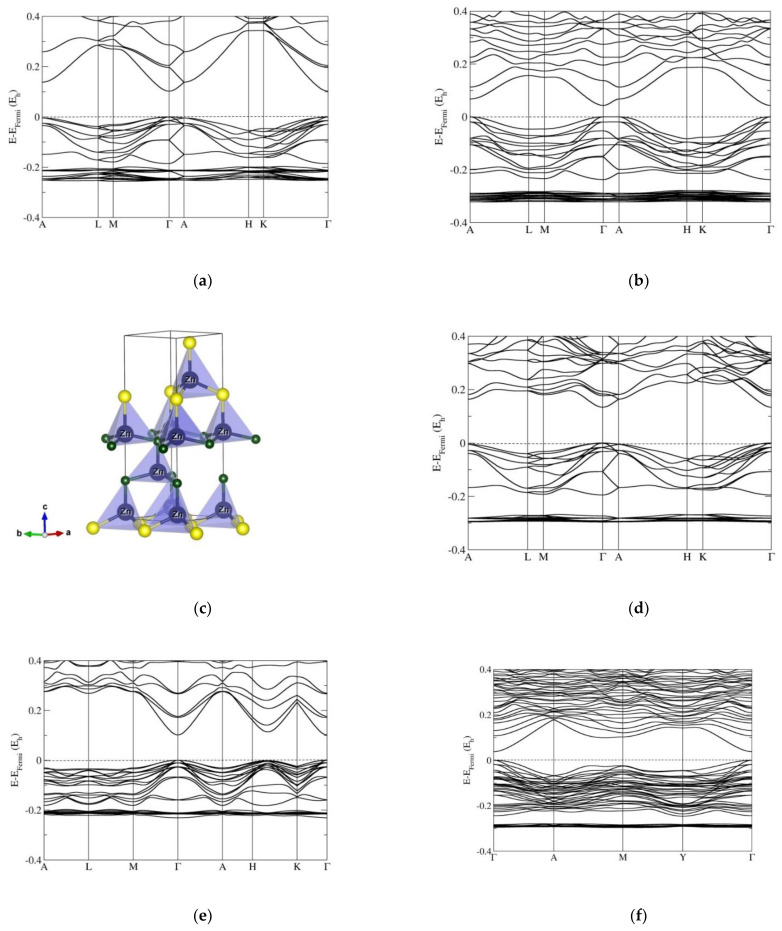

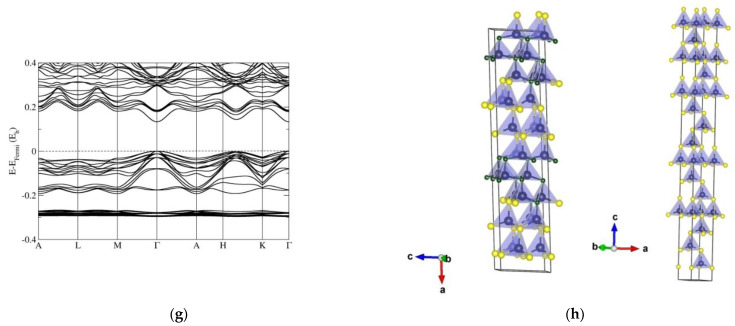
Band structures of the 4H and 15R polytypes for several compositions x, computed using the HSE06 functional: (**a**) pure ZnO with undistorted hexagonal (*P*6_3_*mc*) 4H polytype; (**b**) ZnO_0.5_S_0.5_ compound with slightly distorted 4H structure with trigonal (*P*3*m*1) symmetry; (**d**) pure ZnS compound with undistorted hexagonal (*P*6_3_*mc*) 4H polytype; (**e**) pure ZnO with undistorted rhombohedral (*R*3*m*) 15R polytype; (**f**) ZnO_0.5_S_0.5_ compound with strongly distorted 15R structure with monoclinic (*Cm*) symmetry; (**g**) pure ZnS compound with undistorted rhombohedral 15R polytype. (**c**) depicts the distorted 4H crystal structure for 50% sulfur substitution in ZnO, and (**h**) shows a comparison of the distorted 15R crystal structure for 50% sulfur substitution in ZnO with the structure of pristine ZnS in the 15R modification. Blue, green, and yellow spheres correspond to Zn, O, and S atoms, respectively. Note that the labels of the special points of the Brillouin zones for (**f**) correspond to those of a monoclinic lattice, while all others correspond to those of an hexagonal lattice.

Next, we show band-structure calculations performed on the 4H polytypes in various ZnO/ZnS chemical systems (Figure 3 and Figure S3). The band structure shows a direct band gap at the Γ point of the Brillouin zone in the pristine ZnO and ZnS compounds, but it is not so pronounced as in the 2H or 3C structures. The energy of the TVB along the Γ-A direction of the Brillouin zone is increased with sulfur content x, similar to the case of the 2H structure. Furthermore, such behavior has been observed for various ZnO/ZnS compositions, as well as for many hexagonal polytypic structures (e.g., 2H, 4H, 5H, 6H, and 8H polytypes, Figure 3, Figures S1 and S3). This could be particularly interesting from a point of future applications as direct/indirect ZnO/ZnS semiconductors, with possible applications in photovoltaics (PVs), light-emitting diodes (LEDs), and laser diodes. While the 4H polytype with 25%, 50%, and 75% of sulfur substitution in ZnO is distorted to the trigonal (*P*3*m*1) symmetry, the band gap is strongly reduced in the ZnO_0.75_S_0.25_ compound (HSE06: E_g_ = 0.36 eV; PBE0: E_g_ = 0.98 eV), i.e., we are dealing with a narrow-gap semiconductor, and then the band gap grows again with sulfur content (Figure 3 and Figure S3).

Similarly, we analyze the band structures of the 15R polytypes and related polytypic nanocrystalline structures (Figure 3 and Figure S4). Band structure calculations show a direct band gap at the Γ point of the Brillouin zone in pure ZnO and ZnS compounds. The TVB and BCB along the H-K direction of the Brillouin zone are narrowed (i.e., suggesting the possible development of a secondary gap), observed also in the rhombohedral distorted sphalerite 3C in the ZnO_0.5_S_0.5_ compound. Moreover, this phenomenon has been observed in other rhombohedral polytypes (such as 9R, 12R, and 15R, Figure 3 and Figure S4). In the case of a 20% sulfur content in ZnO, the 15R modification is undistorted and becomes a narrow-gap semiconductor. In the ZnO_0.5_S_0.5_ compound, the 15R polytype is strongly distorted to monoclinic (*Cm*) symmetry, the band gap is reduced, and calculations show a direct band gap at the Γ point of the Brillouin zone, similar to the undistorted 3C phase. Note that the labels of the special points of the Brillouin zones correspond to those of a monoclinic lattice, while all others correspond to those of a hexagonal lattice.

**Figure 4:**
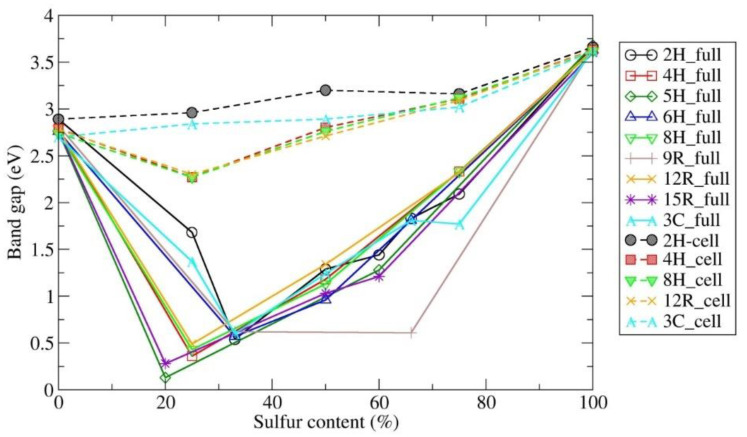
Overview of the computed band gaps of the various polytypes in the ZnO_1−x_S_x_ nanomaterials as a function of sulfur content (x). The calculations were performed for fully relaxed structures (labeled “full”) and relaxed unit cells only (labeled “cell”) using a hybrid HSE06 density functional. Note that the size of the band gap is given in the electron volts (eV).

Finally, in Figure 4, we show an overview of the computed band gaps of the various polytypes in the ZnO_1−x_S_x_ compounds as a function of sulfur content (x) using the hybrid HSE06 functional; the analogous PBE0 calculations are present in the supporting information (c.f., Figure S5) for comparison. Figure 4 and Figure S5 show the same general trend for the PBE0 and the HSE06 functional for the fully relaxed structures. Both of our hybrid calculations (HSE06 and PBE0) show that each of the calculated polytypic structures becomes a narrow-gap semiconductor when adding a small number of sulfur atoms (20–33%), while afterward, the band gap grows again with a further increase in sulfur substitution for oxygen in the original ZnO polytype (Figure 4 and Figure S5). Our first-principles calculations show that the bandgap of ZnO_1−x_S_x_ mixed compounds can be tuned using various polytypes from 0.13 eV to 3.68 eV (e.g., in the 5H polytype) calculated using the HSE06 functional (or from 0.13 eV to 4.29 eV using PBE0, Table S7). However, as mentioned above, additional band structure calculations were performed by fixing the atomic positions in the idealized structure and only relaxing the unit cell parameters. Interestingly, for such relaxed unit cells, only HSE06 calculations show that the band gap of ZnO_1−x_S_x_ mixed compounds can be tuned using various polytypes from 2.27 eV to 3.68 eV, while wurtzite and sphalerite structures types show an even larger band-gap range (Figure 4 and Tables S9 and S10). Our new cell-only calculations are in very good agreement with band-gap measurements of core–shell ZnO/ZnS structures, as well as with previous calculations, as we further analyze below.

## 4. Discussion

The first investigations on ZnO/ZnS solid solutions had taken place in the middle of the past century, reporting that small amounts of oxygen (<1%) can substitute for sulfur in wurtzite and sphalerite, causing changes in the physical properties of both phases [63]. Later, various research groups have studied small nonstoichiometric effects (e.g., doping, vacancies, etc.) on either ZnS or ZnO, causing improvements in semiconducting and optical properties, leading to the deduction of structure–property relationships [64,65,66,67,68,69,70]. Zinc sulfide is the most common natural form of zinc, while ZnO is rarely found, and, therefore, it is not surprising that ZnO/ZnS solid solutions have not been found in nature. However, we would like to point to the work on natural sphalerite crystals from the Trepča mines in Serbia, where twin boundaries deficient in sulfur and enriched in oxygen have been observed [71]. Moreover, we mention the recent study on ultrahigh-temperature sphalerite from Zn-Cd-Se-rich combustion metamorphic marbles from central Jordan, reporting on natural sphalerite as a result of S^2−^ → O^2−^ substitution [72]. These literature data serve as an indication of possibly even naturally occurring ZnO/ZnS crystalline polytypes. Our calculations show that the wurtzite-type may distort to the trigonal *P*3*m*1 space group, and the sphalerite-type structure to the rhombohedral *R*3*m* space group, respectively, which might address some geological questions regarding the mineral matraite or the twinned sphalerite polytype of ZnS [45], and be of relevance regarding the recently experimentally observed defects associated with the high-order oxygen local vibrational modes in ZnS_1−x_O_x_ [73].

On the other hand, polytypism [74], the phenomenon where a chemical compound crystallizes in a variety of periodically repeated layered structures (i.e., the polytypes), widely exists in II–VI compound semiconductors, because of the freedom in the atomic stacking of bilayers consisting of cations and anions [75]. While there exist about 200 experimentally identified stacking variants of ZnS [76], ZnO has only three experimentally known bulk phases: wurtzite and sphalerite under ambient conditions and a rocksalt phase at high pressures. Research on mixed ZnO_1–x_S_x_ compounds and their electronic properties is mainly focused on only two experimentally known phases—wurtzite and sphalerite—therefore finding new modifications with different properties, which might also be tunable by varying the sulfur-to-oxygen ratio that would be highly desirable.

As earlier calculations have shown, mutual alloying of ZnO and ZnS in different structural phases can lead to a dramatic change in the electronic structure, because of the large differences in size and electronegativities of the oxygen and sulfur atoms [77], thus leading to a modification of their electronic and optical properties [78]. This kind of anion substitution leads to a better and more flexible tuning of the electronic band profile than an isovalent cation exchange: while cation substitution mainly shifts the conduction bands, anion substitution affects more strongly the band edges of both the conduction and the valence bands [79]. As the properties of the compound can vary among different structural phases (polymorphs), the relation between electronic structure and crystalline modifications of ZnO/ZnS and finding alternative (meta)stable modifications and theoretical and experimental research of their physical and electronic properties remain an interesting open issue.

Previous calculations of band gaps, together with experimentally measured values, available in the literature for different structure types in pristine ZnO and ZnS, are shown in Tables S2 and S3, respectively. Apart from the ground-state phases—wurtzite and sphalerite (the only ones for which experimental data are available for both ZnO and ZnS)—theoretical references are found only for the 4H and the 6H polytype (in addition to results from our previous work of the ZnO polytypes [45]). Experimental references include average values of all available band-gap measurements [80], band gaps of nanoparticles 8–16 nm in diameter [81], band gaps of 300 nm thick films [82], etc. Some theoretical band gaps that have a good fit with experimental results have been calculated by applying density functional theory (DFT) with LDA and DFT+U for 3C, 6H, 4H, and 2H ZnO crystal structures [83,84]. Similarly, there are calculations using the G_0_W_0_ approach in the literature regarding structural, electronic, and spectral properties of six ZnO polymorphs, including bulk and ultrathin films [85,86]: DFT calculations using the Tran–Blaha-modified Becke–Johnson potential (TB-mBJ) along with the Generalized Gradient Approximation (GGA) of the band gap of various ZnO polymorphs and monolayers [87,88,89], and DFT calculations using an LDA + U functional [67].

Our calculations of the band-gap value for various polytypic structures in the pristine bulk ZnO and ZnS compounds using hybrid PBE0 and HSE06 functionals are shown in the supporting information (Table S4). These calculations are of additional importance for benchmarking due to the small amount of literature data on mixed ZnO/ZnS chemical systems in bulk nanostructured materials (although for a few of the presented ZnS polytypes, there are some band gap calculations, Table S4). Our ab initio results concur with previous experimental and theoretical findings, when existing, where the HSE06 functional agrees better with the ZnO system, and the PBE0 functional shows better agreement with the pure ZnS compound (Tables S2–S4).

A review of the calculated and experimental band-gap values of wurtzite and sphalerite-type ZnO_1−x_S_x_ compounds with different S compositions (x) available in the literature is shown in the supporting information (Table S5). A full potential linearized augmented-plane-wave plus local orbital (FP-L(APW + lo)) approach within Density Functional Theory has been used, as well as a TB-mBJ potential in conjunction with PBE-GGA as an exchange-correlation potential to fit the band-gaps results closer to the experimental results for these highly correlated systems [77]. Among others, experimental measurements of band gaps, such as those on 500–600 nm thick ZnO_1−x_S_x_ (0 ≤ x ≤ 1.0) thin films, have been conducted, and the band-gap energy vs. sulfur content relations have been presented [90]. For both wurtzite and sphalerite types, experimental and theoretical band-gap data have been obtained for various sulfur contents x, without considering the new polytype structures presented within this study, however. We note a wide range of computed band gaps in the literature; in contrast, the HSE06 and PBE0 calculations that we present are quite similar for the whole range of the ZnO_1−x_S_x_ compounds as a function of sulfur composition x.

Results of the present study with calculated band-gap values of wurtzite and sphalerite-type ZnO_1−x_S_x_ compounds with different amounts of sulfur (x) using hybrid PBE0 and HSE06 functionals are presented in the supporting information (Table S6). As mentioned earlier, the PBE0 band gap agrees well with the experimental bulk data for pure ZnO (e.g., for 3C, E_gap-PBE0_ = 3.33 eV compared to the values found in the experiment E_gap-EXP_ = 3.19–3.22 eV [91,92]) and overestimates the pure ZnS band gap (e.g., for 3C, E_gap-PBE0_ = 4.25 eV compared to E_gap-EXP_ = 3.45–3.59 eV [78,93]), while HSE06 underestimates the ZnO band gap (e.g., for 2H, E_gap-HSE_ = 2.89 eV compared to E_gap-EXP_ = 3.10–3.30 eV [82,94]) and yields good agreement with the bulk pure ZnS band gap (e.g., for 2H, E_gap-HSE_ = 3.66 eV compared to E_gap-EXP + THEO_ = 3.60–3.68 eV [82,94]) (Tables S5 and S6). Focusing on the HSE06 functional, we thus expect that our calculations, in general, will slightly underestimate the experimentally observed band gap for bulk modifications, while we expect a slight overestimate for the PBE0 functional.

**Figure 5:**
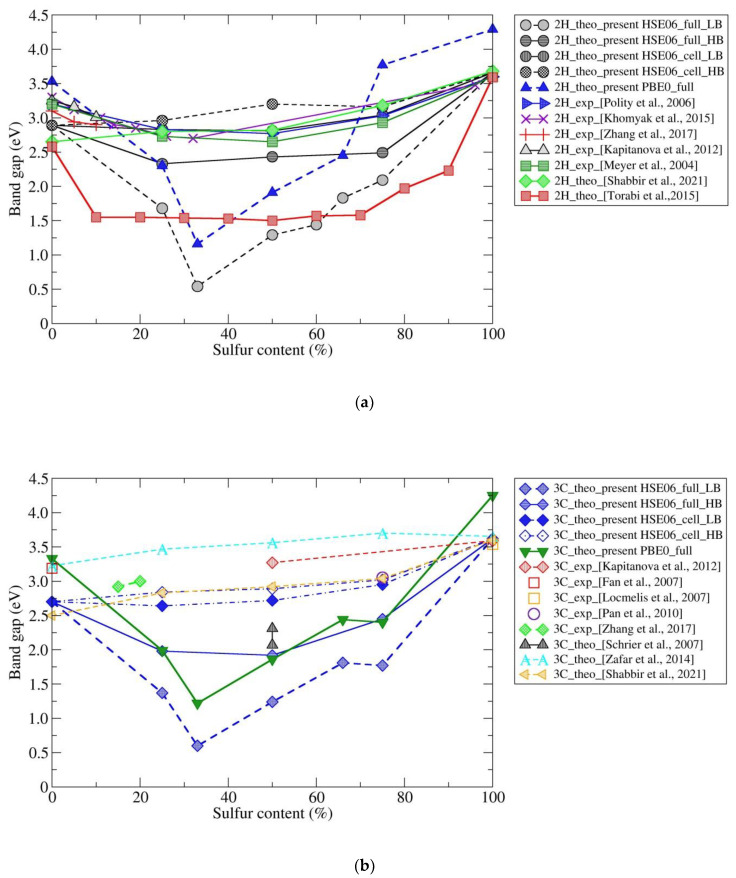
Overview of the computed and measured band gaps size of the (**a**) wurtzite (2H) and (**b**) sphalerite (3C) modifications in the ZnO_1−x_S_x_ compounds as a function of sulfur content (x) using literature data. Note that the size of the band gap is given in electron volts (eV).

Moreover, we have calculated band gaps for the various ZnO/ZnS nanostructures using fixed atomic positions and relaxing the unit cell parameters (which could be used to, e.g., mimic a fixed core/relaxed shell experimental setup in core–shell structures). We note that this structure choice has a dramatic effect on the electronic properties and size of the band gap; furthermore, the results are in very good agreement with band-gap measurements of core–shell ZnO/ZnS nanostructures, as well as with previous calculations using the same kind of structure restrictions. To demonstrate possible trends in the band gap as a function of composition, we present an overview of all the calculated and experimental band-gap values of wurtzite and sphalerite-type ZnO_1−x_S_x_ compounds with different sulfur contents (x) available in the literature (Figure 5a,b). We note the effect of narrowing the band gap with an increase in sulfur (up to 32% of sulfur content), while upon a further increase in sulfur in ZnO, the calculated band gap is also increased in both 2H and 3C, which has also been previously observed (Table S5 and Figure 5a,b). Analyzing the structure–property relationship suggests that the reduction in the band gap may be caused by structural distortions that appear upon introducing sulfur atoms in the 3C and 2H structures of zinc oxide (see Figure 1b and Figure 2b), where the minimum occurs at 33% of sulfur, as both wurtzite 2H and sphalerite 3C distort to unusual trigonal (*P3m1*) symmetry structures for this composition (Figures S1a and S2a). Additional reports of an unusual bandgap bowing in highly mismatched ZnOS alloys [95], and a higher bandgap reduction in hexagonal 2H compared to the cubic 3C ZnO_1−x_S_x_ structure from radio-frequency magnetron sputtering and optical transmission spectroscopy [23,96], support our ab initio results.

Regarding the comparison with the scarce earlier theoretical data for fully or cell-only relaxed structures in various ZnO/ZnS chemical systems, Torabi et al. [97] found a reduction in the band gap down to 1.5 eV for various heterostructures derived from the 2H polytype when adding sulfur to the pure ZnO compound. In contrast, Shabir et al. [77] found a monotonic increase in the band gap for the wurtzite (2H)-based structures as a function of sulfur content. Similarly, Schrier et al. [98] found a reduction in the band gap using the cubic sphalerite (3C) phase, calculating a band gap of 2.07 eV for ZnO/ZnS core/shell-heterostructured nanowires, while Shabir et al. [77] and Zafar et al. [67] found an increase in the band gap.

This large discrepancy is due to the fact that the calculated structures found in the literature by Torabi et al. [97] and Schrier et al. [98] have been fully relaxed with respect to both atom coordinates and cell parameters, which explains why they agree very well with our fully relaxed structures and calculated band gaps (“full”, Figure 5a,b). In contrast, the band gaps calculated by Shabir et al. [77] and Zafar et al. [67] on 2H and 3C phases were obtained using structures with fixed atoms and relaxed unit cells. We have performed such cell-only band structure calculations (labeled “cell”) that concur with their work and previous experimental data on various nanostructures, heterostructures, and heterojunctions (Figure 5a,b). In addition, we have studied the above structures regarding a possible influence of the size and position of sulfur atoms in the structure (Figure 5a,b and Tables S9–S13). Depending on the positions of the sulfur layers, each investigated polytypic structure can appear in four different sulfur layer stacking types (for the largest supercell we have employed in this study), resulting in the lowest band gap size (LB) and the highest band gap size (HB). We note that the greatest influence of the sulfur layer position appeared for the distorted wurtzite structure, causing large variations in the band gap size for a given composition. The highest band (HB) gap values in the sphalerite (3C) phase are influenced by the size of the supercell, while other predicted polytypic structures are barely affected by these changes.

Very interesting are the results of calculations using hybrid HSE06 by Torabi et al. [97], regarding the band gap reduction in ZnO and ZnS by creating layered ZnO/ZnS heterostructures (Figure 5), where the ZnO 2H structure has been substituted by sulfur. The authors suggest mechanisms for such band-gap narrowing by analyzing the DOS in detail. In ZnS, the band gap is found between the highest occupied S 3p level and the lowest unoccupied Zn 4s level, while in ZnO, the gap is between the highest occupied O 2p level and the lowest unoccupied Zn 4s and Zn 4p levels. In the mixed ZnO/ZnS heterostructures, the highest occupied S 3p level in the heterostructures is always higher in energy than the highest occupied O 2p level, and so their band gap is determined by the gap between the highest occupied S 3p level and the lowest unoccupied Zn 4s/4p levels. Furthermore, as the fraction of ZnO in ZnO/ZnS heterostructures increases, the highest occupied S 3p level shifts to higher energies, whereas the lowest unoccupied Zn 4s and Zn 4p levels drift slightly toward lower energies, resulting in a narrowing of the total band gap [97]. The results of that study agree well with our present calculations using the hybrid HSE06 and PBE0 functionals. The present study explores additional possibilities, such as the substitution of ZnO by sulfur, for the sphalerite (3C) structure, allowing a detailed analysis of the band gap vs. sulfur content relationship. In particular, when creating a supercell of 2H and 3C ZnO/ZnS phases, one should take into account the structural distortion caused by sulfur insertion in the initial pure ZnO structure, which affects the electronic properties. In addition, we offer a qualitative and quantitative comparison to the experimental results on the core–shell ZnO/ZnS nanostructures, which seem to suggest relatively thick regions of mixed ZnS-ZnO compounds at the interface, possibly exhibiting one of several of the new crystal polytype nanostructures (4H, 5H, 6H, 8H, 9R, 12R, and 15R) not-yet observed in the experiment.

## 5. Conclusions

Pristine ZnO and ZnS, and, in particular, mixed ZnO_1−x_S_x_ nanomaterials (x = 0.20, 0.25, 0.33, 0.50, 0.60, 0.66, and 0.75) have been investigated on an ab initio level. A major topic of this study is newly identified ZnO/ZnS nanocrystalline polytypes, which provide alternative structural arrangements of ZnO/ZnS compounds on a multi-scale level, and whose structure–property relationship has been investigated. Our ab initio calculations on fully relaxed structures slightly underestimate/overestimate band-gap measurements, depending on the choice of hybrid functional. However, curiously, the band gaps for the (in principle, unstable) fixed atoms-relaxed-cell-only structures show great agreement with present and previous band-gap measurements; this indicates that structural details on the atomic level most likely play a role in stabilizing the structure of the interface between S-rich and O-rich regions of the nanomaterials available in the experiment, which is responsible for the experimentally observed band gaps.

Finally, we report here the results of our theoretical investigation on the electronic structure of many predicted ZnO/ZnS nanocrystalline polytypes calculated using hybrid PBE0 and HSE06 functionals. A large number of band gaps and band structures have been calculated for the pure and mixed ZnO_1−x_S_x_ compounds (x = 0, 0.20, 0.25, 0.33, 0.50, 0.60, 0.66, 0.75, and 1) in the known 2H and 3C structures, as well as in the predicted ZnO/ZnS polytypes 4H, 5H, 6H, 8H, 9R, 12R, and 15R. We would like to highlight the discoveries of alternative polytypic nanomaterials, and that it is possible to influence the size of the band gap by varying structural details of these novel ZnO/ZnS nanomaterials. Moreover, we note the possibility of tuning the band gap as a function of sulfur content, and finally the great importance of the distribution of the sulfur atoms in the ZnO/ZnS structure. In that respect, the greatest influence of the sulfur layer position appeared for the distorted wurtzite structure, causing large variations in the band gap size for a given composition, while other predicted polytypic nanostructures are barely affected by these changes.

The band-structure calculations performed on the predicted 4H polytype in various ZnO/ZnS chemical systems show a direct band gap at the Γ point of the Brillouin zone. The energy of the TVB increases along the Γ-A direction of the Brillouin zone, for various ZnO/ZnS compositions, as well as for many other hexagonal polytype structures (e.g., 2H, 4H, 5H, 6H, and 8H polytypes). Similarly, in the 15R polytype, the TVB and BCB along the H-K direction of the Brillouin zone are narrowed, predicted also in rhombohedral distorted sphalerite 3C in the ZnO_0.5_S_0.5_ compound, and in other rhombohedral polytypes (such as 9R, 12R, and 15R). If we analyze the behavior of the electronic properties as a function of sulfur substitution of ZnO, both our hybrid HSE06 and PBE0 calculations show that each of the predicted polytypic nanostructures becomes a narrow-gap semiconductor for a small amount of sulfur content (20–33%) and that the size of the band gap increases when raising the sulfur content in the ZnO polytype up to the value of the pure ZnS polytype. We conclude that such polytype nanostructures open new perspectives for ZnO/ZnS band-gap engineering, by incorporating the knowledge of the location of the TVB and BCB, to tune the properties of the direct/indirect ZnO/ZnS semiconductors, with possible applications in photovoltaics (PVs), light-emitting diodes (LEDs), laser diodes, nanostructured ceramics, and, finally, for heterojunction solar cells where band alignment is crucial to becoming a narrow-gap semiconductor suitable for possible applications as infrared detectors or in thermoelectrics.

## Figures and Tables

**Table 1:** Calculated unit cell parameters of the newly predicted 4H polytype crystals [43] with various ZnO/ZnS compositions and comparison to the experimental results when existing. Cell parameters are given in nanometers (nm). Local optimizations were performed with the PBE0 and HSE06 hybrid functionals. Note that the 4H type has been observed for pure ZnS.

Chemical Composition		4H Polytype (nm)	
	*PBE0*	*HSE06*	Experiment
**ZnO**	*P*6_3_*mc* (no. 186) *a* = 0.325, *c* = 1.050	*P*6_3_*mc* (no. 186) *a* = 0.325, *c* = 1.051	n/a
**ZnO_0.75_S_0.25_**	*P*3*m*1 (no. 156) *a* = 0.339, *c* = 1.110	*P*3*m*1 (no. 156) *a* = 0.339, *c* = 1.110	n/a
**ZnO_0.5_S_0.5_**	*P*3*m*1 (no. 156) *a* = 0.356, *c* = 1.156	*P*3*m*1 (no. 156) *a* = 0.356, *c* = 1.157	n/a
**ZnO_0.25_S_0.75_**	*P*3*m*1 (no. 156) *a* = 0.372, *c* = 1.209	*P*3*m*1 (no. 156) *a* = 0.372, *c* = 1.210	n/a
**ZnS**	*P*6_3_*mc* (no. 186) *a* = 0.385, *c* = 1.257	*P*6_3_*mc* (no. 186) *a* = 0.386, *c* = 1.259	*P*6_3_*mc* (no. 186) *a* = 0.382, *c* = 1.252 *

* Ref. [61].

**Table 2:** Calculated unit cell parameters of the newly predicted 15R polytype crystals [43] with various ZnO/ZnS compositions and comparison to the experimental results when existing. Cell parameters are given in nanometers (nm). Local optimizations were performed with the PBE0 and HSE06 hybrid functionals. Note that the 15R polymorph has been observed for pure ZnS.

Chemical Composition		15R Polytype (nm)	
	*PBE0*	*HSE06*	Experiment
**ZnO**	*R*3*mH* (no. 160) *a* = 0.325, *c* = 3.944	*R*3*mH* (no. 160) *a* = 0.325, *c* = 3.946	n/a
**ZnO_0.8_S_0.2_**	*R*3*mH* (no. 160) *a* = 0.336, *c* = 4.107	*R*3*mH* (no. 160) *a* = 0.336, *c* = 4.109	n/a
**ZnO_0.5_S_0.5_**	*Cm* (no. 8) *a* = 2.895, *b* = 0.356, *c* = 0.615, β = 94.04	*Cm* (no. 8) *a* = 2.896, b = 0.356, *c* = 0.616, β = 94.04	n/a
**ZnO_0.4_S_0.6_**	*R*3*mH* (no. 160) *a* = 0.363, *c* = 4.422	*R*3*mH* (no. 160) *a* = 0.363, *c* = 4.425	n/a
**ZnS**	*R*3*mH* (no. 160) *a* = 0.385, *c* = 4.719	*R*3*mH* (no. 160) *a* = 0.386, *c* = 4.740	*R3mH* (no. 160) *a* = 0.382, *c* = 4.680 *

* Ref. [62].

## Data Availability

The data presented in this study are available on request from the corresponding author.

## Supplementary Materials

The following supporting information can be downloaded at: https://www.mdpi.com/article/10.3390/nano12091595/s1, Supporting Tables, Table S1: Calculated unit cell parameters of the pure ZnS and ZnO predicted polytypes, and experimental results when existing; Table S2: Calculated and experimental band-gap value references of different polytypic structures in the pristine ZnO compound; Table S3: Calculated and experimental band-gap value references of different polytypic structures in the pristine ZnS compound; Table S4: Calculated band gaps of different polytype structures in pristine ZnO and ZnS compounds using hybrid PBE0, HSE06, and B3LYP functionals; Table S5: Calculated and experimental band-gap value references of wurtzite and sphalerite type ZnO_1−x_S_x_ compounds with different sulfur content (x); Table S6: Calculated band gap of the wurtzite (a) and the sphalerite (b) type ZnO_1−x_S_x_ compounds with different sulfur content (x) using hybrid PBE0 and HSE06 functionals; Table S7: Calculated band gaps of the predicted 4H (a), 5H (b), 6H (c), and 8H (d) polytypes of ZnO_1−x_S_x_ compounds with different sulfur content (x) using hybrid PBE0 and HSE06 functionals; Table S8: Calculated band gaps of predicted 9R (a), 12R (b), and 15R (c) polytypes of ZnO_1−x_S_x_ compounds with different sulfur content (x) using hybrid PBE0 and HSE06 functionals; Table S9: Calculated band gap of the wurtzite type ZnO_1−x_S_x_ compounds with different sulfur content (x) and four types (V1-V4) depending on the sulfur layer position (see Table S14). Calculations were performed using the HSE06 hybrid functional; Table S10: Calculated band gap of the sphalerite type ZnO_1−x_S_x_ compounds with different sulfur content (x), and two different sizes of the supercell (small/large). The four types (V1–V4) with different sulfur layer positions (see Table S14) yielded essentially the same results. Calculations were performed using the HSE06 hybrid functional; Table S11: Calculated band gap of the predicted 4H polytype ZnO_1−x_S_x_ compounds with different sulfur content (x) and four types (V1–V4) depending on the sulfur layer positions (see Table S14). Calculations were performed using the HSE06 hybrid functional; Table S12: Calculated band gap of the predicted 8H polytype ZnO_1−x_S_x_ compounds with different sulfur content (x) and four types (V1–V4) depending on the sulfur layer positions (see Table S14). Calculations were performed using the HSE06 hybrid functional; Table S13: Calculated band gap of the predicted 12R polytype ZnO_1−x_S_x_ compounds with different sulfur content (x) and four types (V1–V4) depending on the sulfur layer position (see table S14). Calculations were performed using the HSE06 hybrid functional; Table S14: Possible arrangements of layers of sulfur atoms in the experimentally observed wurtzite (2H) and sphalerite (3C) structures, and predicted 4H, 8H, and 12R polytypic structures (four different types), which were employed for evaluating energies and band gaps, for three different compositions ZnO_0.75_S_0.25_ (a), ZnO_0.5_S_0.5_ (b), and ZnO_0.25_S_0.75_ (c). The column entries of S/O for each type indicate the sequence of sulfur and oxygen layers in the supercell, which define the different types of arrangements; Supporting Figures, Figure S1: Band structures of the wurtzite (2H) phase in the: (a) ZnO_0.66_S_0.33_ composition with slightly distorted 2H structure with trigonal (*P3m1*) symmetry; (b) ZnO_0.25_S_0.75_ compound with strongly distorted monoclinic (*Pm*) 2H phase. Calculations were performed using the HSE06 functional; Figure S2: Band structures of the sphalerite (3C) phase in the: (a) ZnO_0.66_S_0.33_; and (b) ZnO_0.33_S_0.66_ composition with highly distorted 3C structure with trigonal (*P3m1*) symmetry. Calculations were performed using the HSE06 functional; Figure S3: Band structures of the 4H phase in the: a) ZnO_0.75_S_0.25_; and b) ZnO_0.25_S_0.75_ composition with slightly distorted 4H polytypic structure with trigonal (*P3m1*) symmetry. Calculations were performed using the HSE06 functional; Figure S4: Band structures of the 15R phase in the: (a) ZnO_0.80_S_0.20_; and (b) ZnO_0.40_S_0.60_ composition with slightly distorted 15R polytypic structure with trigonal (*P3m1*) symmetry. Calculations were performed using the HSE06 functional; Figure S5: Overview of the computed band gaps of the various polytypes in the ZnO_1−x_S_x_ system as a function of sulfur content (x). The calculations were performed using a hybrid PBE0 functional. Note that the band gap is given in electron volts (eV). References [99,100,101,102,103,104,105,106,107,108,109,110,111,112,113,114,115,116,117,118,119,120,121] are cited in the supplementary materials.
