# Supplementary material for: Band Gap Engineering of Newly Discovered ZnO/ZnS Polytypic Nanomaterials
Source: Nanomaterials (Basel). 2022 May 8;12(9):1595. doi: 10.3390/nano12091595 (PMC9101784; doi:10.3390/nano12091595)
Supplement: Supplementary file 1 [file nanomaterials-12-01595-s001.zip › nanomaterials-1694919-supplementary.pdf]

nanomaterials

Supplementary Materials

Band Gap Engineering of Newly Discovered ZnO/ZnS Polytypic Nanomaterials

Dejan Zagorac [1,2,*], Jelena Zagorac [1,2], Milan Pejić [1,2], Branko Matović [1,2] and Johann Christian Schön [3,*]

1 Materials Science Laboratory, Institute of Nuclear Sciences "Vinča", University of Belgrade, 11000 Belgrade, Serbia; jelena@vinca.rs (J.Z.); milan.pejic@yahoo.com (M.P.); mato@vinca.rs (B.M.)
2 Center for Synthesis, Processing and Characterization of Materials for Application in the Extreme Conditions "Cextreme Lab", Institute of Nuclear Sciences, University of Belgrade, 11001 Belgrade, Serbia
3 Nanoscale Science Department, Max Planck Institute for Solid State Research, 70569 Stuttgart, Germany
* Correspondence: dzagorac@vinca.rs (D.Z.); c.schoen@fkf.mpg.de (J.C.S.)

Supporting Tables

Table S1. Calculated unit cell parameters of the pure ZnS and ZnO predicted polytypes, and experimental results when existing. Cell parameters are given in nm. Local optimizations were performed with the PBE0 and HSE06 hybrid functionals.

Structure type	ZnS (nm)			ZnO (nm)		
Space group	*PBE0*	*HSE*	Experiment	*PBE0*	*HSE*	Experiment
**Wurtzite (2H)** **$P6_3mc$ (no. 186)**	*a* = 0.386, *c* = 0.628	a = 0.386, c = 0.629	*a* = 0.382, *c* = 0.626 [99]	*a* = 0.326, *c* = 0.520	a = 0.327, c = 0.520	a = 0.325, c = 0.521 [103]
**Sphalerite (3C)** ***F-43m* (no. 216)**	*a* = 0.545	*a* = 0.546	*a* = 0.540 [5,100]	*a* = 0.458	*a* = 0.458	*a* = 0.4595 [104]
**4H** **$P6_3mc$ (no. 186)**	*a* = 0.385, *c* = 1.257	*a* = 0.386, *c* = 1.259	*a* = 0.382, *c* = 1.252 [61]	*a* = 0.325, *c* = 1.050	*a* = 0.325, *c* = 1.051	n/a
**5H** ***P3m1* (no. 156)**	*a* = 0.385, *c* = 1.570	*a* = 0.385, *c* = 1.571	n/a	*a* = 0.325, *c* = 1.314	*a* = 0.325, *c* = 1.315	n/a
**6H** **$P6_3mc$ (no. 186)**	*a* = 0.385, *c* = 1.886	*a* = 0.386, *c* = 1.888	*a* = 0.382, *c* = 1.874 [101]	*a* = 0.325, *c* = 1.580	*a* = 0.325, *c* = 1.580	n/a
**8H** **$P6_3mc$ (no. 186)**	*a* = 0.385, *c* = 2.516	*a* = 0.386, *c* = 2.518	*a* = 0.382, *c* = 2.496 [102]	*a* = 0.324, *c* = 2.109	*a* = 0.324, *c* = 2.110	n/a
**9R** ***R3mH* (no. 160)**	*a* = 0.385, *c* = 2.830	*a* = 0.386, *c* = 2.832	*a* = 0.382, *c* = 2.808 [62]	*a* = 0.325, *c* = 2.356	*a* = 0.326, *c* = 2.357	n/a
**12R** ***R3mH* (no. 160)**	*a* = 0.385, *c* = 3.773	*a* = 0.386, *c* = 3.776	*a* = 0.382, *c* = 3.744 [62]	*a* = 0.325, *c* = 3.150	*a* = 0.325, *c* = 3.151	n/a
**15R** ***R3mH* (no. 160)**	*a* = 0.385, *c* = 4.719	*a* = 0.386, *c* = 4.740	*a* = 0.382, *c* = 4.680 [62]	*a* = 0.325, *c* = 3.944	*a* = 0.325, *c* = 3.946	n/a

Table S2. Calculated and experimental band-gap value references of different polytypic structures in the pristine ZnO compound.

**ZnO structure type**	**Band-gap value E_g (eV)**	
	**Theoretical (calculated)**	**Experimental**
Wurtzite (2H)	2.901 [87]	3.44 [105]
	3.479 [83]	3.40 [107]
	0.8 [108]	3.27 (3.10–3.40) [80]
	~0.75 [106]	3.25(2) [93]
	2.68 [109]	3.1 [81]
	2.65 [77]	3.20 [82,84,111]
	3.31 [85]	3.30 [90]
4H	3.425 [83]	n/a
	~0.7 [106]	
6H	3.410 [83]	n/a
	~0.68 [106]	
Sphalerite (3C)	2.679 [87]	3.19 [91]
	3.381 [83]	3.22 [92]
	~0.65 [106]	
	3.225 [67]	
	3.3 [112]	
	3.18 [85]	
	2.5 [77]	
	2.50 [110]	

Table S3. Calculated and experimental band-gap value references of different polytypic structures in the pristine ZnS compound.

ZnS structure type	Band-gap value E_g (eV)	
	**Theoretical (calculated)**	**Experimental**
Wurtzite (2H)	3.88 [75]	3.75 (3.60–3.91) [80]
	4.08 [116]	3.86 [113]
	3.98 [117]	3.91 [114]
	3.68 [77]	3.60 [82,94,111]
	3.68 [119]	3.62 [90]
4H	3.84 [75]	
6H	3.83 [75]	
Sphalerite (3C)	3.80 [75]	3.82 [115]
	3.98 [117]	3.59(3) [93]
	3.80 [118]	3.54 [78]
	3.655 [67]	3.45 [120]
	3.94 [112]	
	3.60 [77]	
	3.70 [110]	

Table S4. Calculated band gaps of different polytype structures in pristine ZnO and ZnS compounds using hybrid PBE0, HSE06, and B3LYP functionals.

Structure type	ZnO E_g (eV)			ZnS E_g (eV)	
	**HSE06**	**PBE0**	**B3LYP**	**HSE06**	**PBE0**
Wurtzite (2H)	2.89	3.53	3.21[a]	3.66	4.29
4H	2.79	3.42	3.15[a]	3.63	4.26
5H	2.76	3.39	3.12[a]	3.68	4.29
6H	2.76	3.39	3.11[a]	3.63	4.26
8H	2.74	3.37	3.09[a]	3.62	4.26
9R	2.82	3.45	3.16[a]	3.64	4.27
12R	2.78	3.42	3.08[a]	3.63	4.26
15R	2.77	3.40	3.13[a]	-	-
Sphalerite (3C)	2.70	3.33	3.08[a]	3.62	4.25

Source: [a] ref. [45].

Table S5. Calculated and experimental band-gap value references of wurtzite and sphalerite type $ZnO_{1-x}S_x$ compounds with different sulfur content (x). Calculations are performed for partially relaxed strucutres ("cell-only" relaxations).

**$ZnO_{1-x}S_x$**		**Band-gap value E_g (eV)**									
**Composition (x)**		**x = 0 (ZnO)**	**x = 0.05**	**x = 0.1**	**x = 0.11–0.19**	**x = 0.25**	**x = 0.26–0.32**	**x = 0.5**	**x = 0.75**	**x = 0.96**	**x = 1 (ZnS)**
Wurtzite (2H)	Exp.	3.25(2) [93]	3.16(1) [93]	3.01(2) [93]	3.00 [*, [90]]	2.83 [111]	2.73 [$, [26]]	2.77 [111]	3.03 [111]	3.48 [26]	3.60 [82,94,111]
		3.1 [81]	2.94 [81]	2.90 [81]	2.90 [**, [90]]	2.73 [82,94]	2.70 [$$, [26]]	2.65 [82,94]	2.93 [82,94]		3.62 [90]
		3.20 [82,94,111]	3.12 [90]		2.85 [***, [90]]						
		3.30 [90]									
	Calc.	2.65 [77]<br>2.68 [109]				2.80 [77]		2.82 [77]	3.18 [121]		3.68 [77]<br>3.68 [119]
Sphalerite (3C)	Exp.	3.19 [91]<br>3.22 [92]			2.92 [#, [81]]<br>3.0 [##, [81]]			3.27(2) [93]	3.05 [121]		3.59(3) [93]<br>3.54 [78]<br>3.45 [120]
	Calc.	3.225 [67]<br>3.3 [119]<br>2.5 [77]<br>2.50 [110]				3.468 [67]<br>2.83 [77]<br>2.60 [110]		3.561 [67]<br>2.92 [77]<br>2.80 [110]<br>2.31 [98]	3.702 [67]<br>3.04 [77]<br>3.00 [110]		3.655 [67]<br>3.94 [112]<br>3.60 [77]<br>3.70 [110]

[*] x = 0.11; [**] x = 0.14; [***] x = 0.19; [$] x = 0.26; [$$] x = 0.32; [#]x = 0.15; [##]x = 0.20.

Table S6. Calculated band gap of the wurtzite (a) and the sphalerite (b) type $ZnO_{1-x}S_x$ compounds with different sulfur content (x) using hybrid PBE0 and HSE06 functionals.

(a)

Chemical composition	Wurtzite (2H) E_g (eV)	
	HSE06	PBE0
ZnO	2.89	3.53
$ZnO_{0.75}S_{0.25}$	1.68	2.30
$ZnO_{0.66}S_{0.33}$	0.54	1.16
$ZnO_{0.5}S_{0.5}$	1.29	1.91
$ZnO_{0.33}S_{0.66}$	1.83	2.45
$ZnO_{0.25}S_{0.75}$	2.09	3.77
ZnS	3.66	4.29

(b)

Chemical composition	Sphalerite (3C) E_g (eV)	
	HSE06	PBE0
ZnO	2.70	3.33
$ZnO_{0.75}S_{0.25}$	1.37	1.99
$ZnO_{0.66}S_{0.33}$	0.60	1.22
$ZnO_{0.5}S_{0.5}$	1.24	1.86
$ZnO_{0.33}S_{0.66}$	1.81	2.44
$ZnO_{0.25}S_{0.75}$	1.77	2.40
ZnS	3.62	4.25

Table S7. Calculated band gaps of the predicted 4H (a), 5H (b), 6H (c), and 8H (d) polytypes of $ZnO_{1-x}S_x$ compounds with different sulfur content (x) using hybrid PBE0 and HSE06 functionals. .

(a)

**Chemical composition**	**4H polytype E_g (eV)**	
	**HSE06**	**PBE0**
ZnO	2.79	3.42
$ZnO_{0.75}S_{0.25}$	0.36	0.98
$ZnO_{0.5}S_{0.5}$	1.18	1.80
$ZnO_{0.25}S_{0.75}$	2.33	2.96
ZnS	3.63	4.26

(b)

**Chemical composition**	**5H polytype E_g (eV)**	
	**HSE06**	**PBE0**
ZnO	2.76	3.39
$ZnO_{0.8}S_{0.2}$	0.13	0.13
$ZnO_{0.4}S_{0.6}$	1.28	1.28
ZnS	3.68	4.29

(c)

**Chemical composition**	**6H polytype E_g (eV)**	
	**HSE06**	**PBE0**
ZnO	2.89	3.53
$ZnO_{0.66}S_{0.33}$	0.54	1.16
$ZnO_{0.5}S_{0.5}$	1.29	1.91
$ZnO_{0.33}S_{0.66}$	1.83	2.45
ZnS	3.66	4.29

(d)

**Chemical composition**	**8H polytype E_g (eV)**	
	**HSE06**	**PBE0**
ZnO	2.74	3.37
$ZnO_{0.75}S_{0.25}$	0.43	1.05
$ZnO_{0.5}S_{0.5}$	1.13	1.74
$ZnO_{0.25}S_{0.75}$	2.33	2.96
ZnS	3.62	4.26

Table S8. Calculated band gaps of predicted 9R (a), 12R (b), and 15R (c) polytypes of $ZnO_{1-x}S_x$ compounds with different sulfur content (x) using hybrid PBE0 and HSE06 functionals. .

(a)

**Chemical composition**	**9R polytype E_g (eV)**	
	**HSE06**	**PBE0**
ZnO	2.82	3.45
$ZnO_{0.66}S_{0.33}$	0.62	1.24
$ZnO_{0.33}S_{0.66}$	0.61	1.24
ZnS	3.64	4.27

(b)

**Chemical composition**	**12R polytype E_g (eV)**	
	**HSE06**	**PBE0**
ZnO	2.78	3.42
$ZnO_{0.75}S_{0.25}$	0.49	1.11
$ZnO_{0.5}S_{0.5}$	1.34	1.96
$ZnO_{0.25}S_{0.75}$	2.32	2.95
ZnS	3.63	4.26

(c)

**Chemical composition**	**15R polytype E_g (eV)**	
	**HSE06**	**PBE0**
ZnO	2.77	3.40
$ZnO_{0.8}S_{0.2}$	0.28	0.91
$ZnO_{0.5}S_{0.5}$	1.03	1.65
$ZnO_{0.4}S_{0.6}$	1.21	1.89
ZnS	3.62	4.25

Table S9. Calculated band gap of the wurtzite type $ZnO_{1-x}S_x$ compounds with different sulfur content (x) and four types (V1-V4) depending on the sulfur layer position (see Table S14). Calculations were performed using the HSE06 hybrid functional. .

(a)

Type of S	Wurtzite (2H) E_g (eV) $ZnO_{0.75}S_{0.25}$			
	**Full**		Cell	
	**Band**	Total energy	Band	Total energy
V1 (I)	1.68	−1935.0609	2.83	−1935.0488
V2 (II)	1.63	−1935.0597	2.83	−1935.0488
V3 (III)	2.33	−1935.0616	2.96	−1935.0492
V4 (IV)	2.33	−1935.0616	2.96	−1935.0492

(b)

Type of S	Wurtzite (2H) E_g (eV) $ZnO_{0.5}S_{0.5}$			
	**Full**		Cell	
	**Band**	Total energy	Band	Total energy
V1 (I)	1.29	−2015.7847	2.81	−2015.7742
V2 (II)	2.43	−2015.7919	3.20	−2015.7749
V3 (III)	2.02	−2015.7877	2.96	−2015.7746
V4 (IV)	1.96	−2015.7880	2.99	−2015.7746

(c)

Type of S	Wurtzite (2H) E_g (eV) $ZnO_{0.25}S_{0.75}$			
	**Full**		Cell	
	**Band**	Total energy	Band	Total energy
V1 (I)	2.10	−2096.5198	3.16	−2096.5110
V2 (II)	2.28	−2096.5196	3.13	−2096.5110
V3 (III)	2.13	−2096.5189	3.04	−2096.5109
V4 (IV)	2.49	−2096.5205	3.15	−2096.5110

Table S10. Calculated band gap of the sphalerite type $ZnO_{1-x}S_x$ compounds with different sulfur content (x), and two different sizes of the supercell (small/large). The four types (V1–V4) with different sulfur layer positions (see Table S14) yielded essentially the same results. Calculations were performed using the HSE06 hybrid functional.

(a)

	**Sphalerite (3C) E_g (eV) $ZnO_{0.75}S_{0.25}$**			
**Type of S**	**Full**		Cell	
	**Band**	Total energy	Band	Total energy
V1–V4(small)	1.98	−1935.0603	2.84	−1935.0490
V1–V4(large)	1.36	−1935.0583	2.64	−1935.0482

(b)

	**Sphalerite (3C) E_g (eV) $ZnO_{0.5}S_{0.5}$**			
**Type of S**	**Full**		Cell	
	**Band**	Total energy	Band	Total energy
V1–V4(small)	1.92	−2015.7878	2.89	−2015.7751
V1–V4(large)	1.24	−2015.7845	2.72	−2015.7740

(c)

	**Sphalerite (3C) E_g (eV) $ZnO_{0.25}S_{0.75}$**			
**Type of S**	**Full**		Cell	
	**Band**	Total energy	Band	Total energy
V1–V4(small)	2.45	−2096.5198	3.02	−2096.5108
V1–V4(large)	1.77	−2096.5182	2.95	−2096.5108

Table S11. Calculated band gap of the predicted 4H polytype $ZnO_{1-x}S_x$ compounds with different sulfur content (x) and four types (V1–V4) depending on the sulfur layer positions (see Table S14). Calculations were performed using the HSE06 hybrid functional. .

a)

Type of S	4H polytype E_g (eV) $ZnO_{0.75}S_{0.25}$			
	Full		Cell	
	Band	Total energy	Band	Total energy
V1	0.36	-1935.0589	2.27	-1935.0472
V2	0.37	-1935.0589	2.27	-1935.0471
V3	0.43	-1935.0590	2.25	-1935.0471
V4	0.42	-1935.0590	2.25	-1935.0471

b)

Type of S	4H polytype E_g (eV) $ZnO_{0.5}S_{0.5}$			
	Full		Cell	
	Band	Total energy	Band	Total energy
V1	1.18	-2015.7859	2.62	-2015.7728
V2	1.33	-2015.7846	2.80	-2015.7741
V3	1.21	-2015.7846	2.69	-2015.7740
V4	1.17	-2015.7859	2.62	-2015.7728

c)

Type of S	4H polytype E_g (eV) $ZnO_{0.25}S_{0.75}$			
	Full		Cell	
	Band	Total energy	Band	Total energy
V1	2.33	-2096.5175	3.11	-2096.5104
V2	2.09	-2096.5172	3.00	-2096.5103
V3	2.10	-2096.5172	3.00	-2096.5103
V4	2.33	-2096.5175	3.11	-2096.5104

Table S12. Calculated band gap of the predicted 8H polytype $ZnO_{1-x}S_x$ compounds with different sulfur content (x) and four types (V1-V4) depending on the sulfur layer positions (see Table S14). Calculations were performed using the HSE06 hybrid functional. .

a)

Type of S	8H polytype E_g (eV) $ZnO_{0.75}S_{0.25}$ Full		Cell	
	**Band**	**Total energy**	**Band**	**Total energy**
V1	0.43	-1935.0589	2.27	-1935.0471
V2	0.32	-1935.0584	2.22	-1935.0470
V3	0.34	-1935.0589	2.25	-1935.0471
V4	0.28	-1935.0585	2.18	-1935.0469

b)

Type of S	8H polytype E_g (eV) $ZnO_{0.5}S_{0.5}$ Full		Cell	
	**Band**	**Total energy**	**Band**	**Total energy**
V1	1.13	-2015.7849	2.63	-2015.7728
V2	1.02	-2015.7848	2.57	-2015.7727
V3	1.02	-2015.7847	2.61	-2015.7733
V4	1.27	-2015.7846	2.76	-2015.7740

c)

Type of S	8H polytype E_g (eV) $ZnO_{0.25}S_{0.75}$ Full		Cell	
	**Band**	**Total energy**	**Band**	**Total energy**
V1	2.33	-2096.5174	3.12	-2096.5103
V2	2.10	-2096.5172	3.02	-2096.5103
V3	1.86	-2096.5172	2.85	-2096.5102
V4	1.83	-2096.5173	2.88	-2096.5103

Table S13. Calculated band gap of the predicted 12R polytype $ZnO_{1-x}S_x$ compounds with different sulfur content (x) and four types (V1-V4) depending on the sulfur layer position (see table S14). Calculations were performed using the HSE06 hybrid functional.

a)

12R polytype E_g (eV) $ZnO_{0.75}S_{0.25}$				
**Type of S**	**Full**		**Cell**	
	**Band**	**Total energy**	**Band**	**Total energy**
V1	0.49	-1935.0595	2.30	-1935.0474
V2	0.47	-1935.0589	2.31	-1935.0471
V3	0.31	-1935.0584	2.22	-1935.0469
V4	0.31	-1935.0590	2.21	-1935.0472

(b)

12R polytype E_g (eV) $ZnO_{0.5}S_{0.5}$				
**Type of S**	**Full**		**Cell**	
	**Band**	**Total energy**	**Band**	**Total energy**
V1	1.34	-2015.7860	2.71	-2015.7730
V2	1.01	-2015.7849	2.54	-2015.7728
V3	-	-	2.63	-2015.7729
V4	1.13	-2015.7850	-	-

-optimization failed

c)

12R polytype E_g (eV) $ZnO_{0.25}S_{0.75}$				
**Type of S**	**Full**		**Cell**	
	**Band**	**Total energy**	**Band**	**Total energy**
V1	2.32	-2096.5174	3.09	-2096.5104
V2	2.09	-2096.5173	3.00	-2096.5104
V3	2.09	-2096.5172	3.01	-2096.5103
V4	2.31	-2096.5173	3.11	-2096.5103

Table S14. Possible arrangements of layers of sulfur atoms in the experimentally observed wurtzite (2H) and sphalerite (3C) structures, and predicted 4H, 8H, and 12R polytypic structures (four different types), which were employed for evaluating energies and band gaps, for three different compositions $ZnO_{0.75}S_{0.25}$ (a), $ZnO_{0.5}S_{0.5}$ (b), and $ZnO_{0.25}S_{0.75}$ (c). The column entries of S/O for each type indicate the sequence of sulfur and oxygen layers in the supercell, which define the different types of arrangements.

a)

$ZnO_{0.75}S_{0.25}$			
**Type I**	**Type II**	**Type III**	**Type IV**
S	S	S	S
S	O	O	O
O	S	O	O
O	O	S	O
O	O	O	S
O	O	O	O
O	O	O	O
O	O	O	O

b)

$ZnO_{0.5}S_{0.5}$			
**Type I**	**Type II**	**Type III**	**Type IV**
S	S	S	S
S	S	O	S
S	O	S	S
S	O	O	O
O	S	S	S
O	S	O	O
O	O	S	O
O	O	O	O

c)

$ZnO_{0.25}S_{0.75}$			
**Type I**	**Type II**	**Type III**	**Type IV**
S	S	S	S
S	S	S	S
S	S	S	S
S	S	S	O
S	S	O	S
S	O	S	S
O	S	S	S
O	O	O	O

Supporting Figures

(a) **(b)**

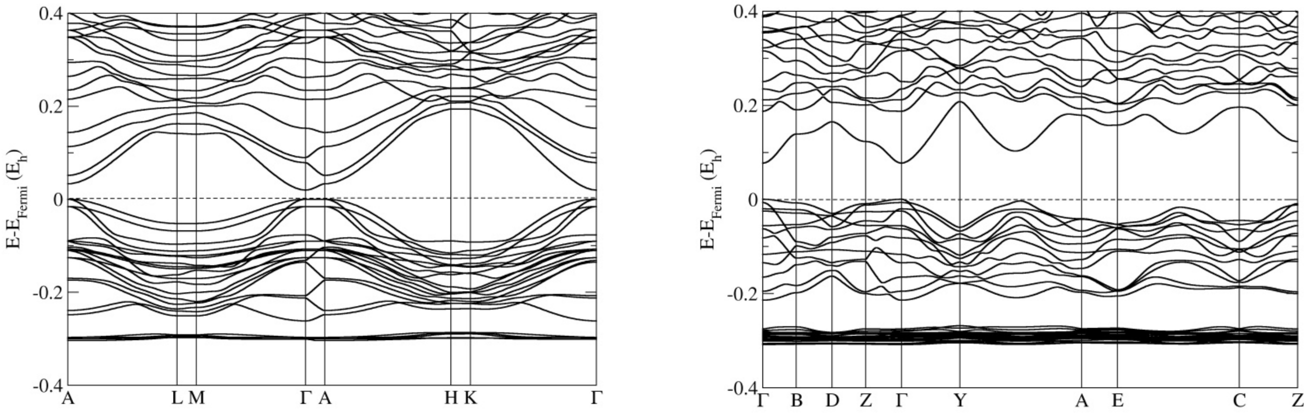

Figure S1. Band structures of the wurtzite (2H) phase in the: (**a**) $ZnO_{0.66}S_{0.33}$ composition with slightly distorted 2H structure with trigonal (*P3m1*) symmetry; (**b**) $ZnO_{0.25}S_{0.75}$ compound with strongly distorted monoclinic (*Pm*) 2H phase. Calculations were performed using the HSE06 functional.

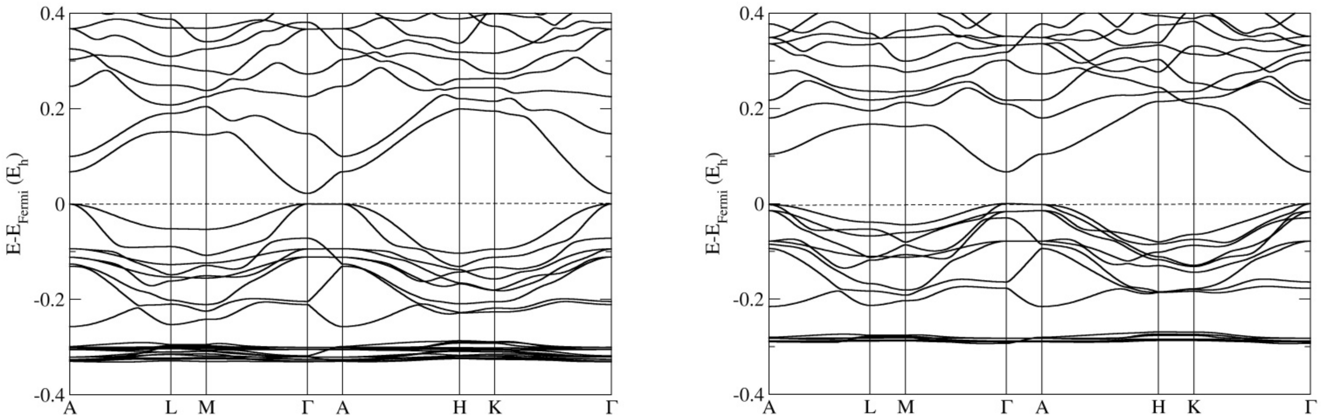

(**a**) (**b**)

Figure S2. Band structures of the sphalerite (3C) phase in the: (**a**) $ZnO_{0.66}S_{0.33}$; and (**b**) $ZnO_{0.33}S_{0.66}$ composition with highly distorted 3C structure with trigonal (*P3m1*) symmetry. Calculations were performed using the HSE06 functional.

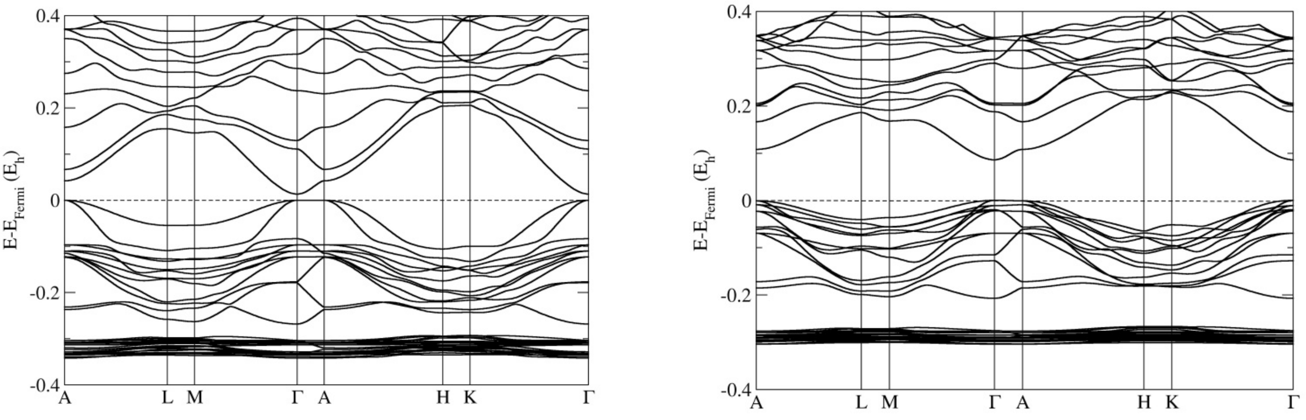

(**a**) (**b**)

Figure S3. Band structures of the 4H phase in the: (**a**) $ZnO_{0.75}S_{0.25}$; and (**b**) $ZnO_{0.25}S_{0.75}$ composition with slightly distorted 4H polytypic structure with trigonal (*P3m1*) symmetry. Calculations were performed using the HSE06 functional.

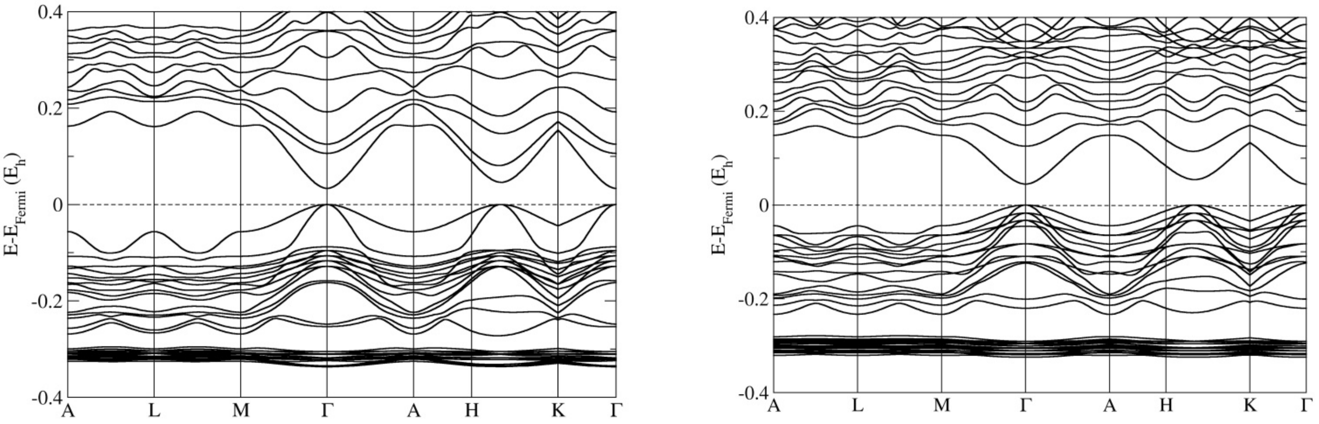

(**a**) (**b**)

Figure S4. Band structures of the 15R phase in the: (**a**) $ZnO_{0.80}S_{0.20}$; and (**b**) $ZnO_{0.40}S_{0.60}$ composition with slightly distorted 15R polytypic structure with trigonal (*P3m1*) symmetry. Calculations were performed using the HSE06 functional.

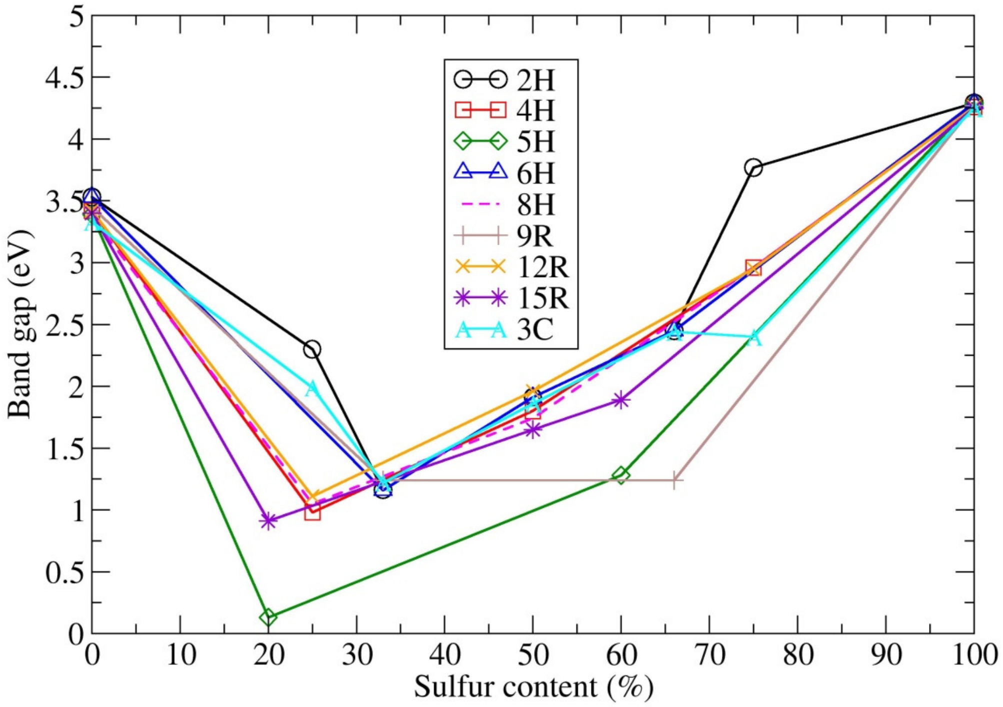

Figure S5. Overview of the computed band gaps of the various polytypes in the $ZnO_{1-x}S_x$ system as a function of sulfur content (x). The calculations were performed using a hybrid PBE0 functional. Note that the band gap is given in electron volts (eV).
